# Supplementary material for: Multiscale core-periphery structure in a global liner shipping network
Source: Sci Rep. 2019 Jan 23;9:404. doi: 10.1038/s41598-018-35922-2 (PMC6344524; doi:10.1038/s41598-018-35922-2)
Supplement: Supplementary file 1 — Supplementary Information: Multiscale core-periphery structure in global liner shipping networks [file 41598_2018_35922_MOESM1_ESM.pdf]

Multiscale core-periphery structure in a global liner shipping network

Sadamori Kojaku, Mengqiao Xu, Haoxiang Xia and Naoki Masuda

(a) Sample 1

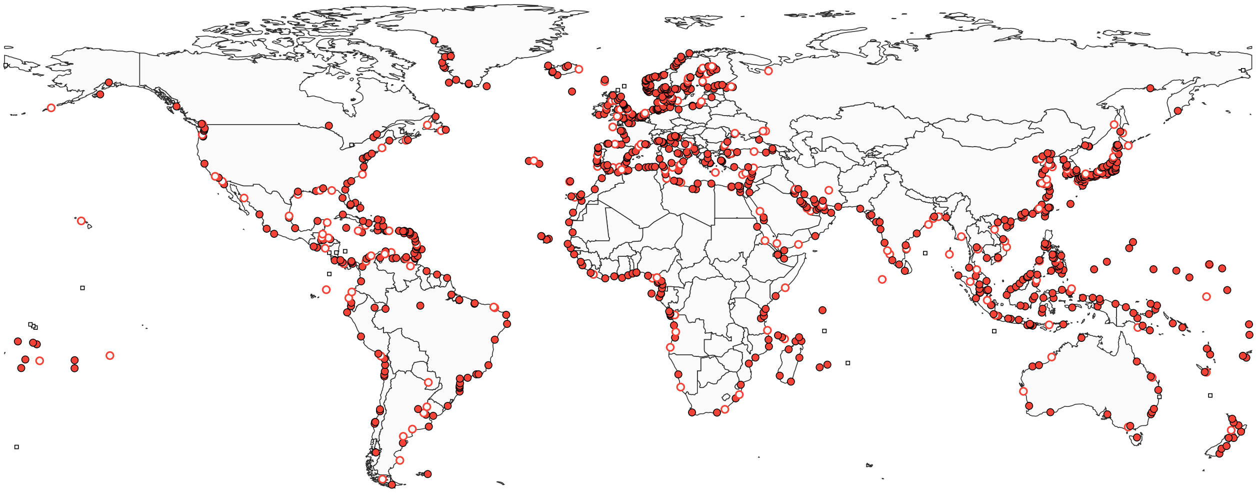

(b) Sample 2

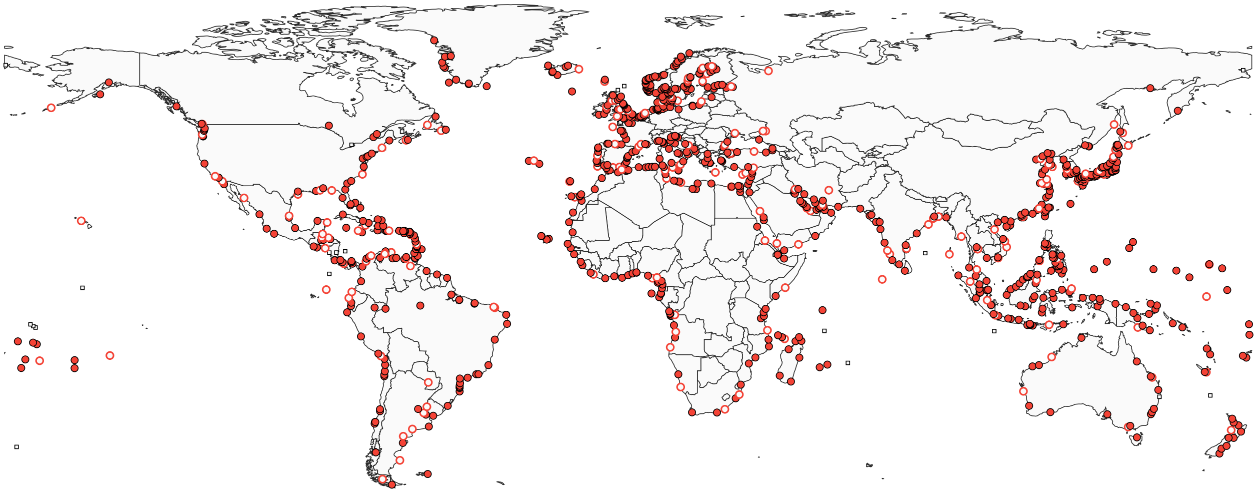

(c) Sample 3

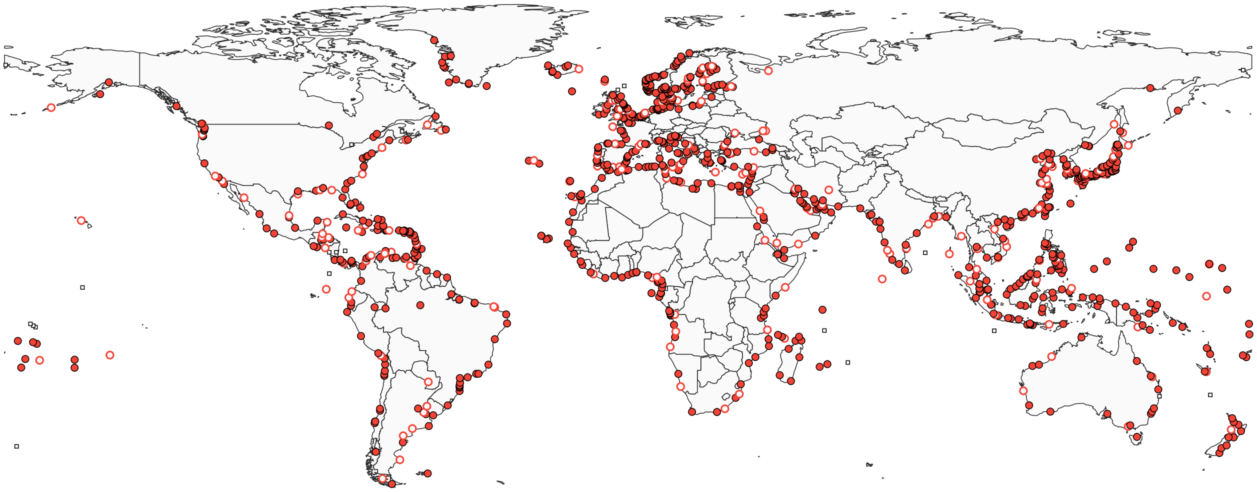

Supplementary Figure S1. CP structure detected at resolution $\gamma = 0.01$ (1st–3rd samples). The filled and open circles indicate the core and peripheral ports, respectively. The open squares indicate the ports belonging to the insignificant CP pair. The colour of each port indicates the ID of the CP pair.

(a) Sample 4

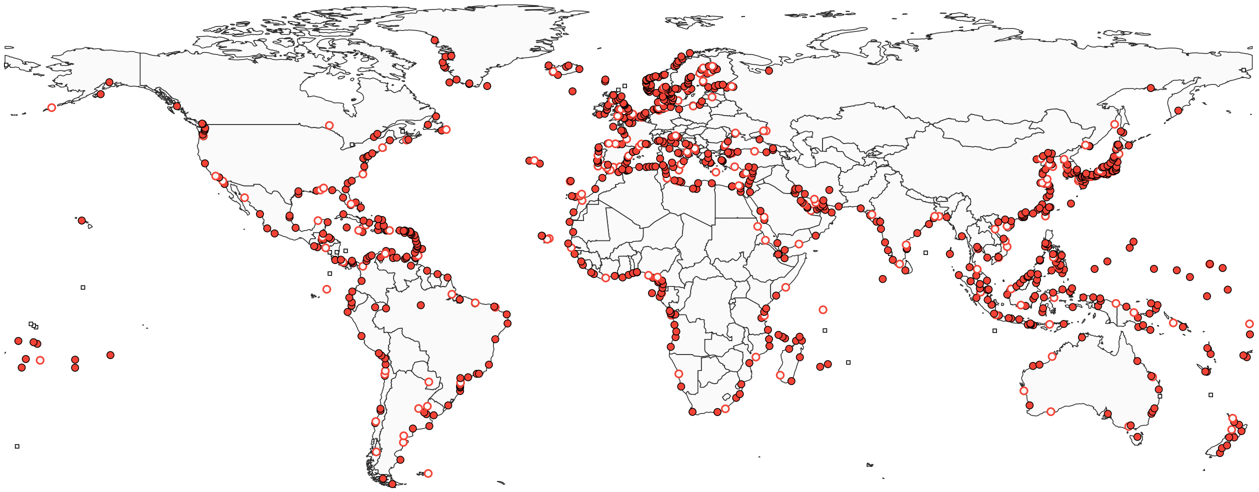

(b) Sample 5

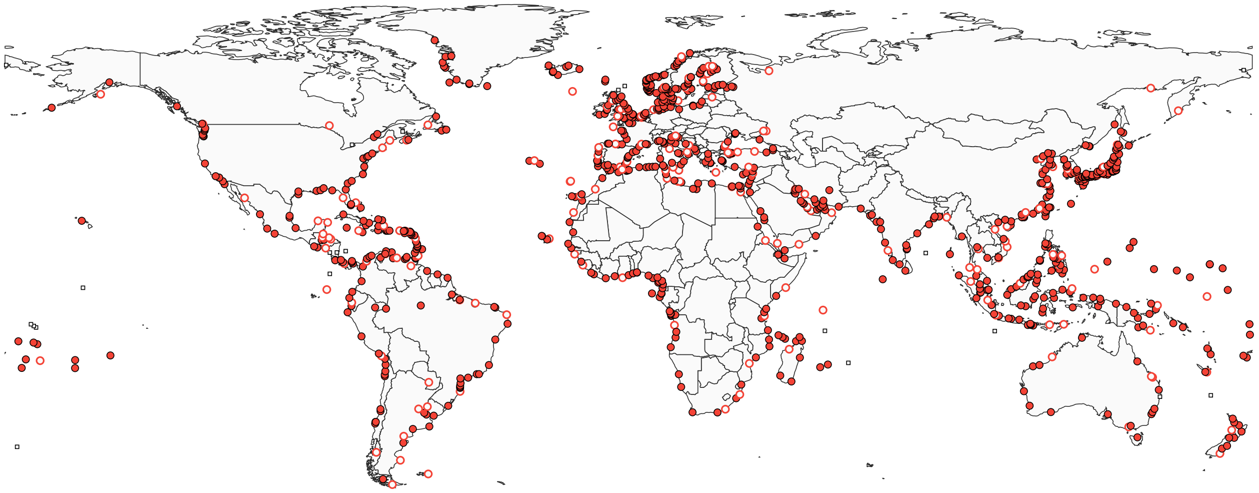

(c) Sample 6

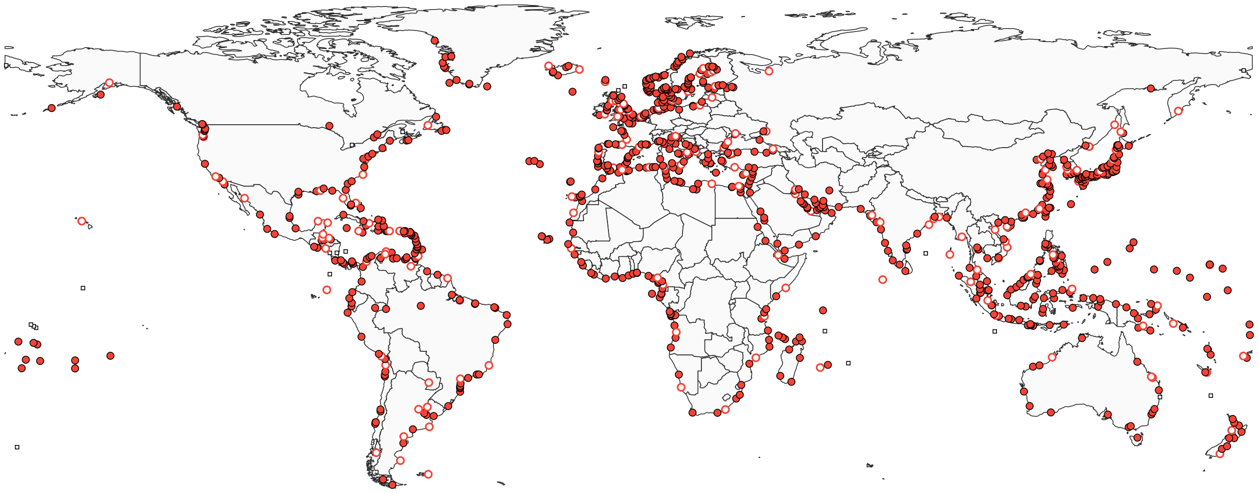

Supplementary Figures S1 (continued). 4th–6th samples.

(a) Sample 1

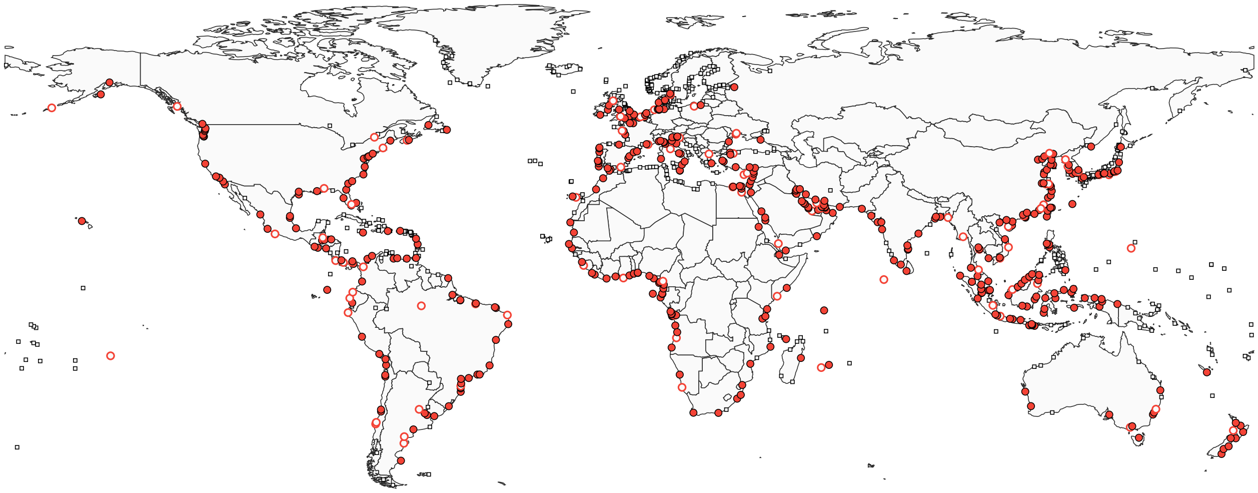

(b) Sample 2

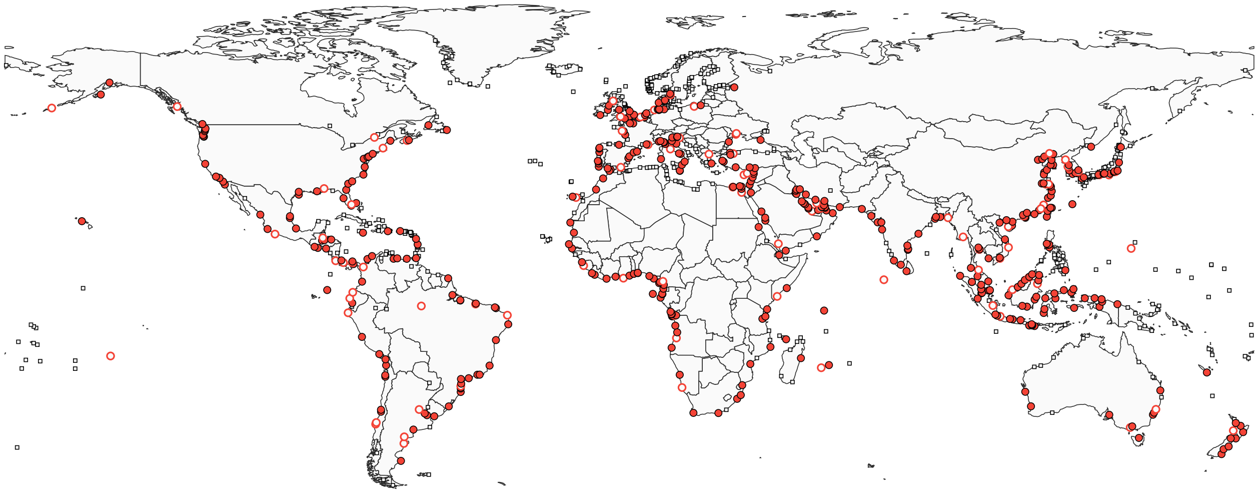

(c) Sample 3

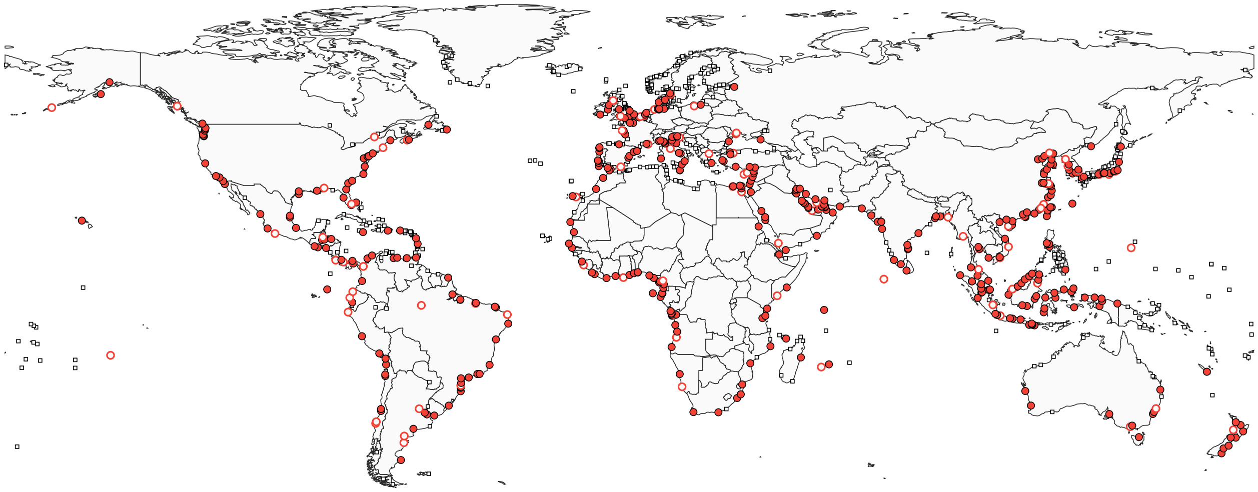

Supplementary Figure S2. CP structure detected at resolution $\gamma = 0.1$ (1st–3rd samples).

(a) Sample 4

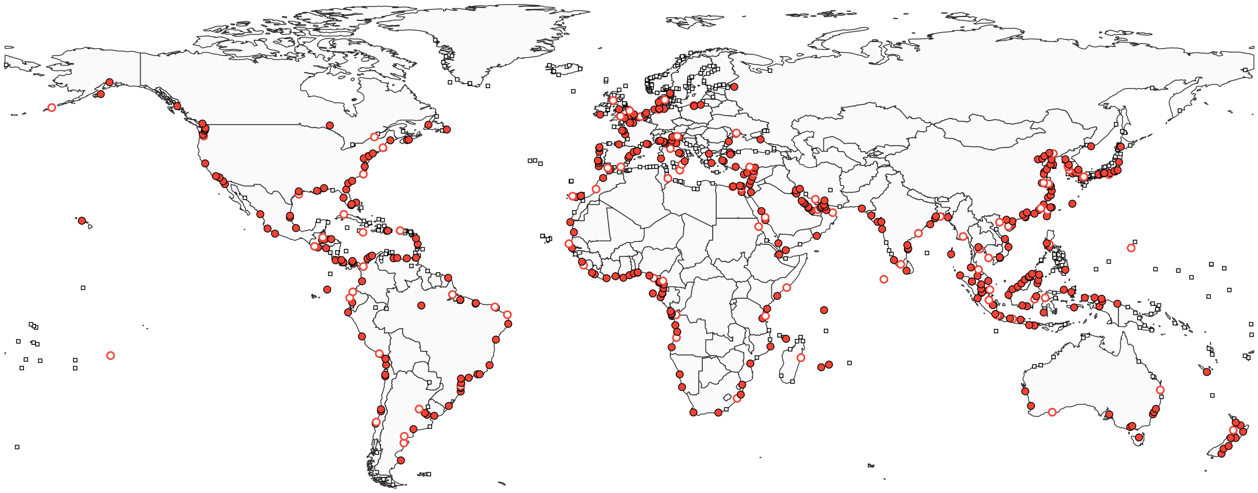

(b) Sample 5

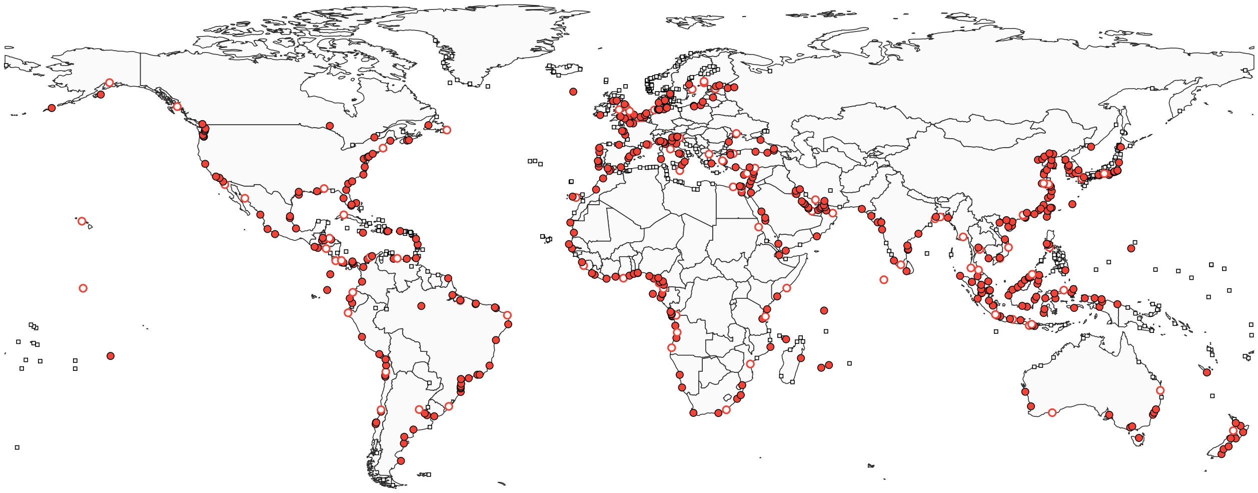

(c) Sample 6

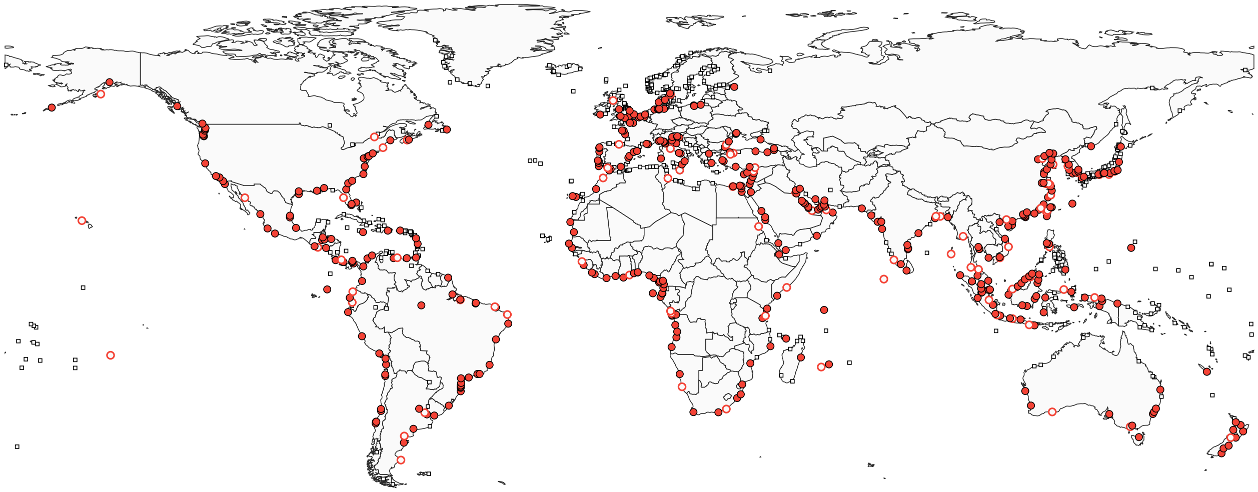

Supplementary Figures S2 (continued). 4th–6th samples.

(a) Sample 1

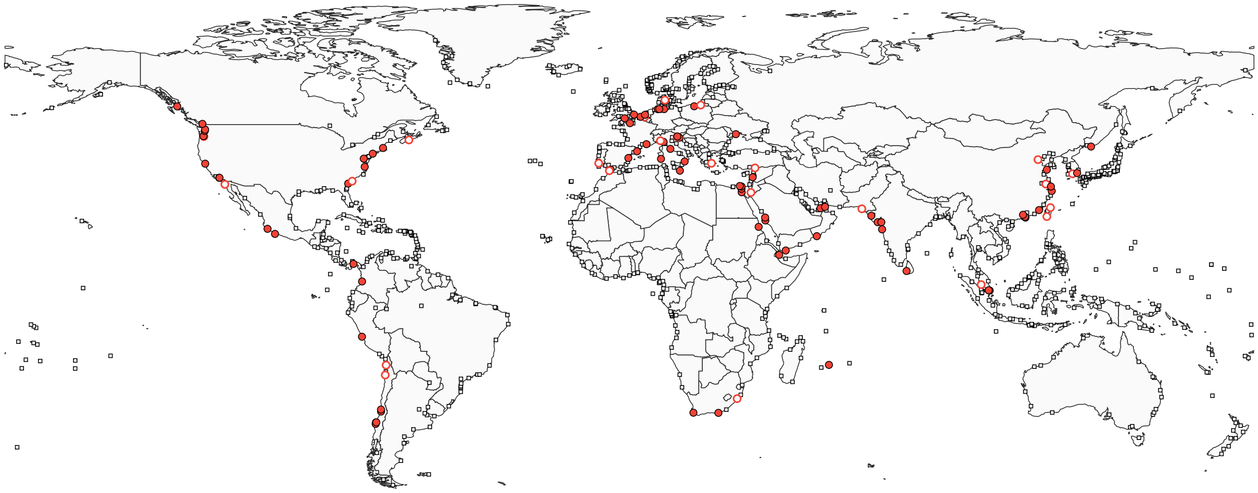

(b) Sample 2

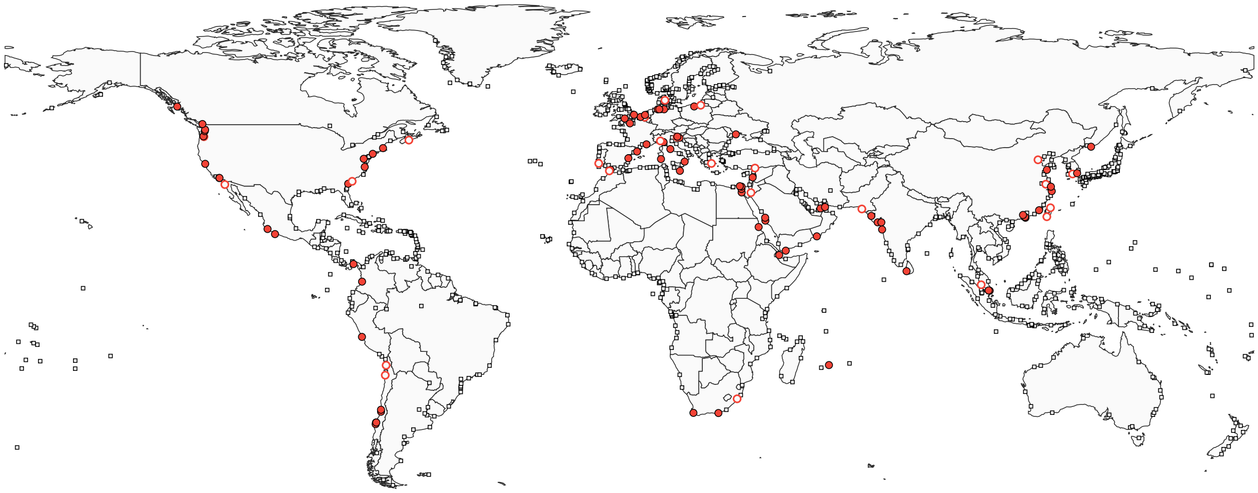

(c) Sample 3

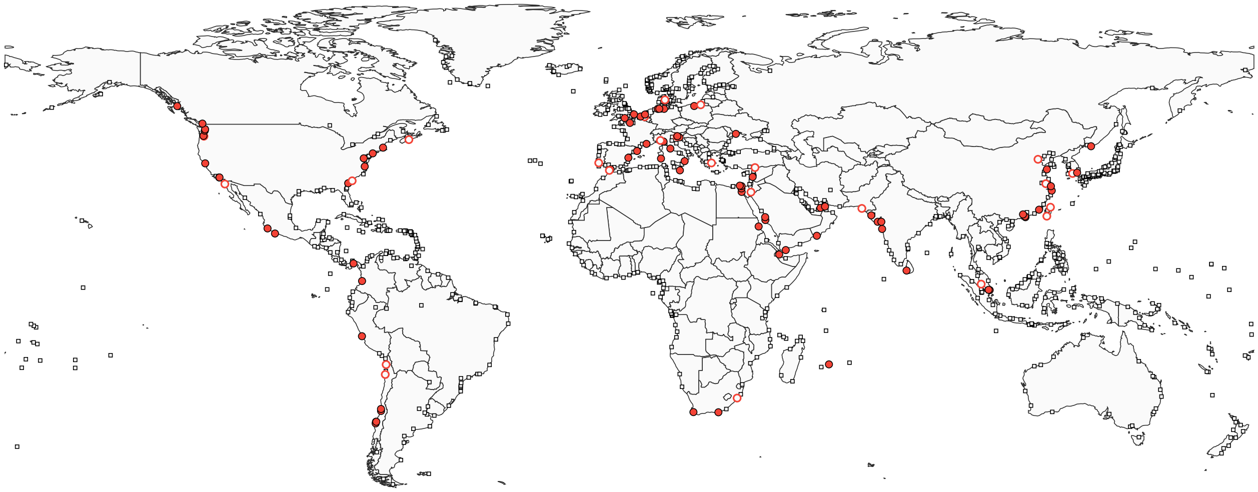

Supplementary Figure S3. CP structure detected at resolution $\gamma = 1.9$ (1st–3rd samples).

(a) Sample 4

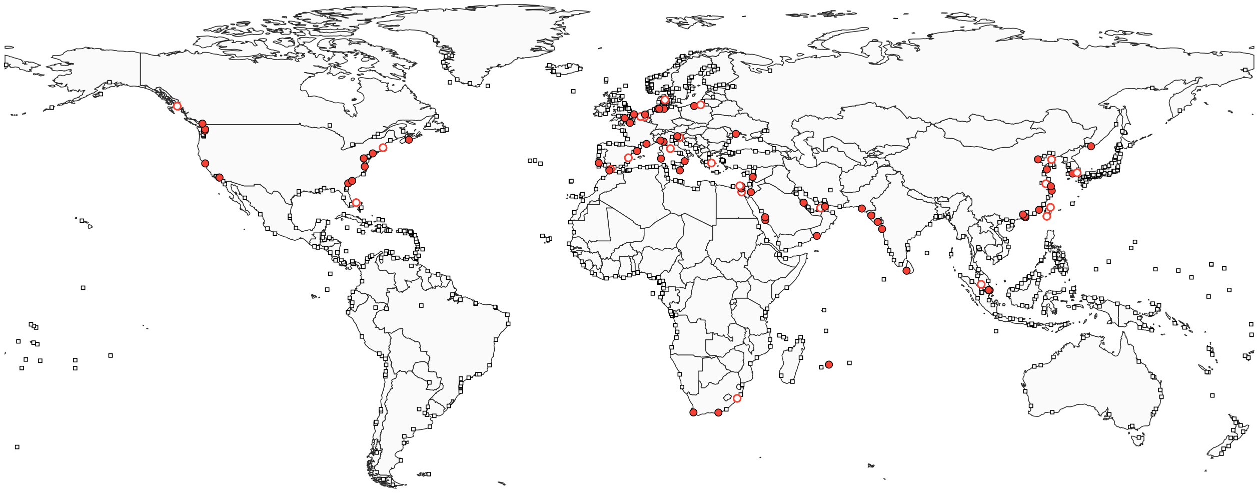

(b) Sample 5

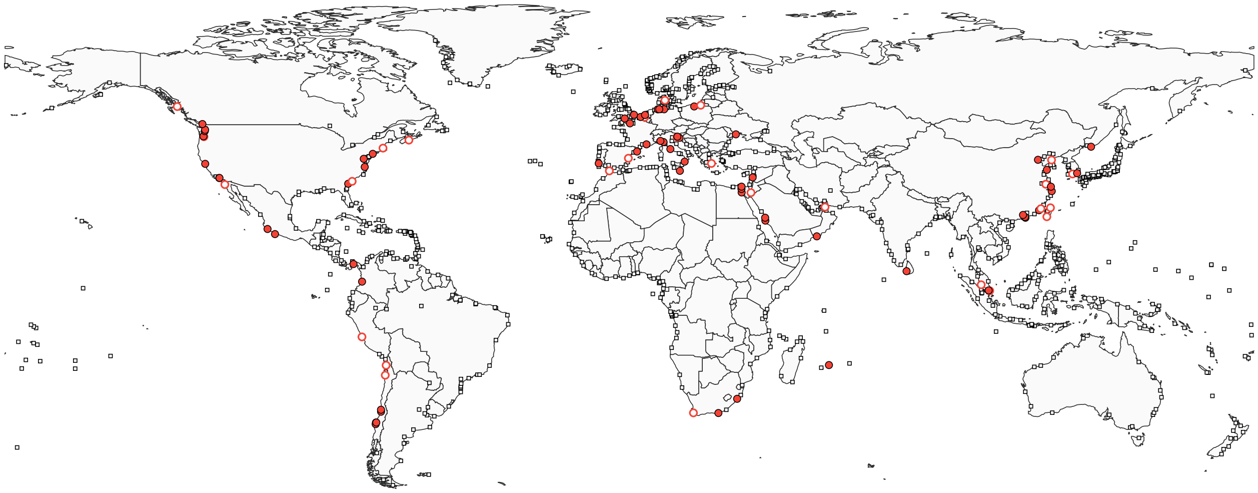

(c) Sample 6

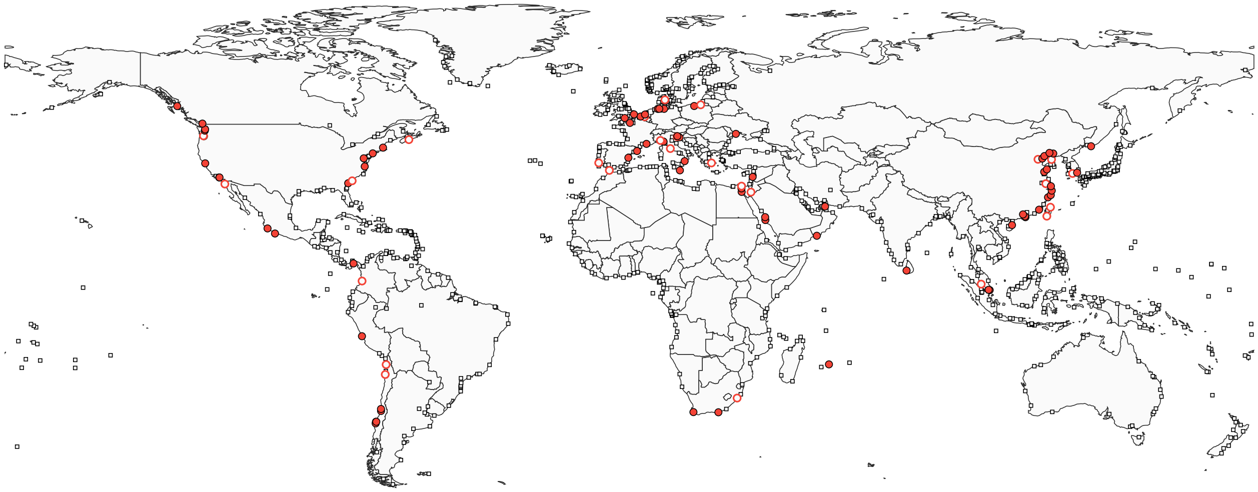

Supplementary Figures S3 (continued). 4th–6th samples.

(a) Sample 1

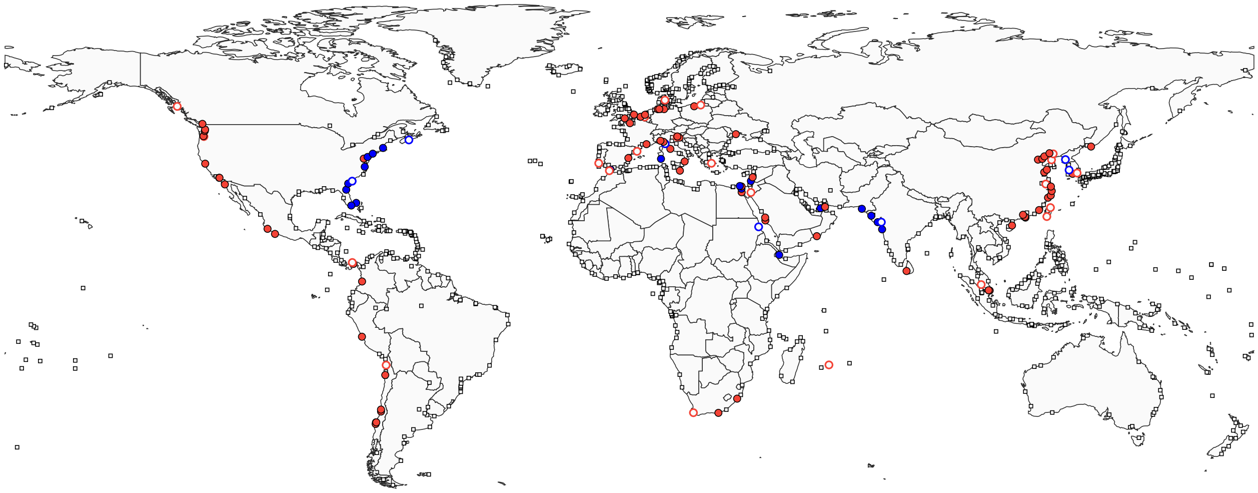

(b) Sample 2

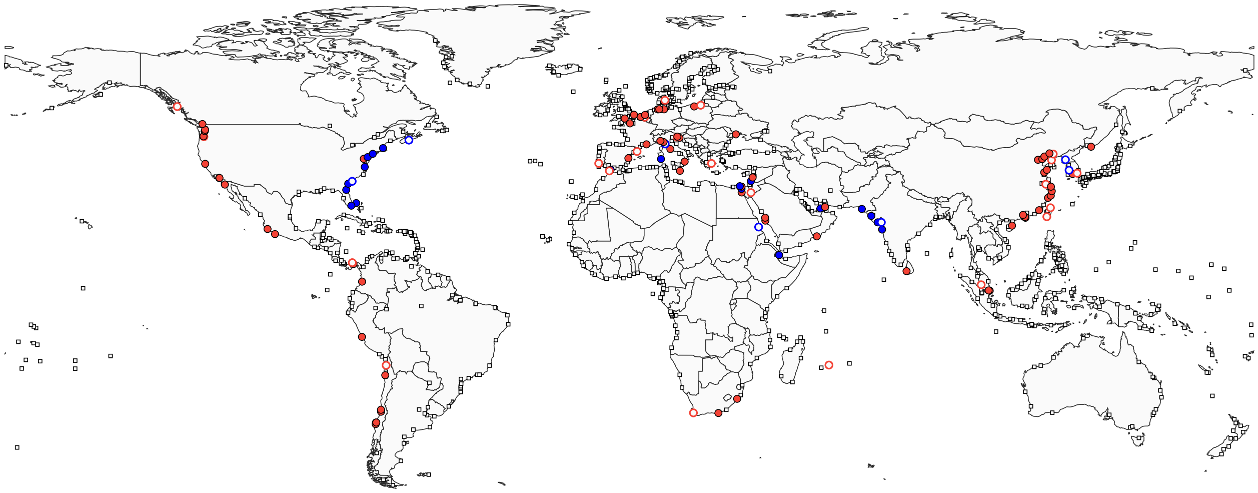

(c) Sample 3

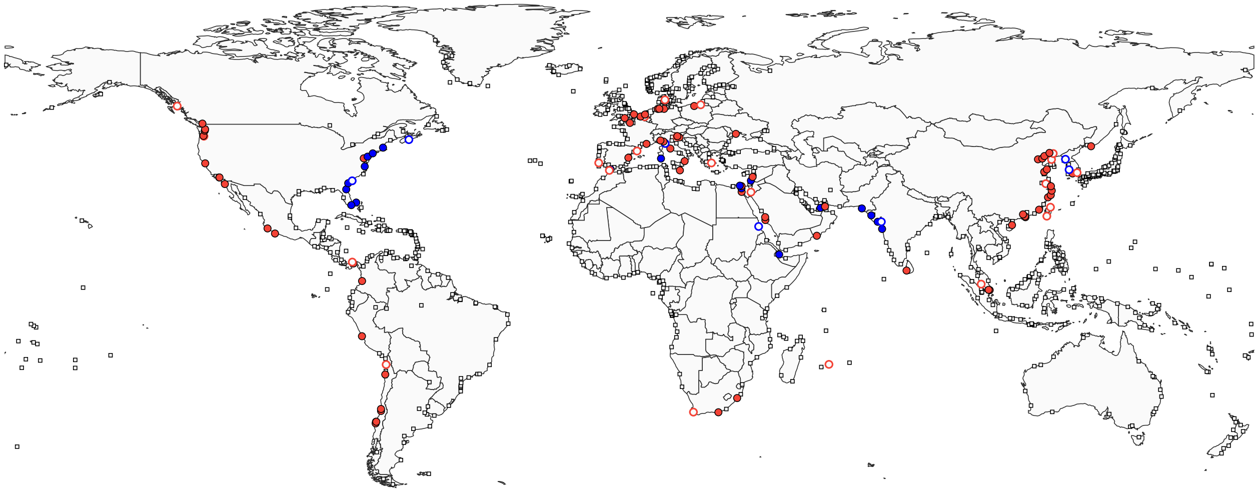

Supplementary Figure S4. CP structure detected at resolution $\gamma = 2.0$ (1st–3rd samples).

(a) Sample 4

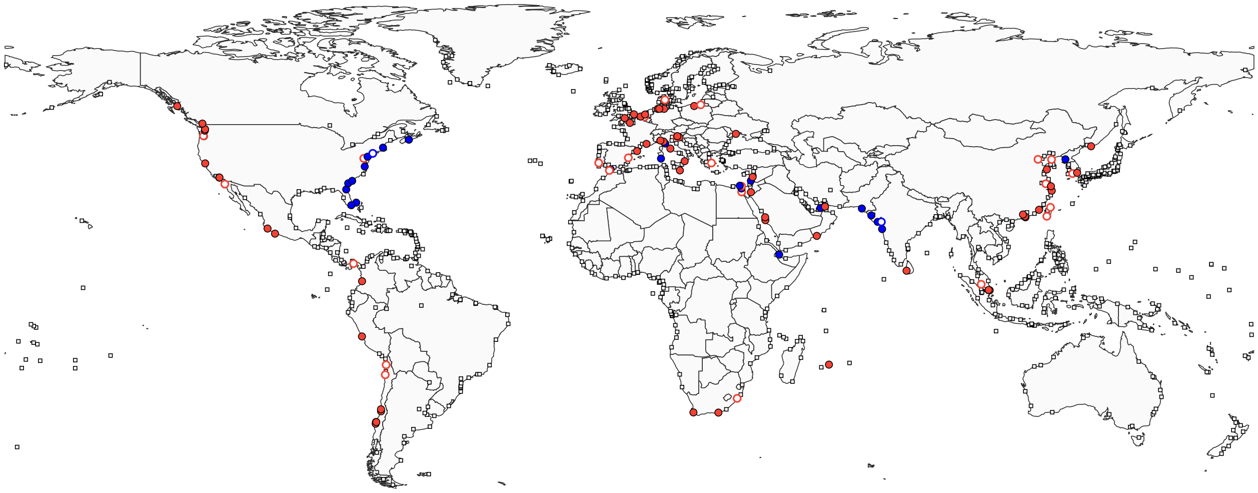

(b) Sample 5

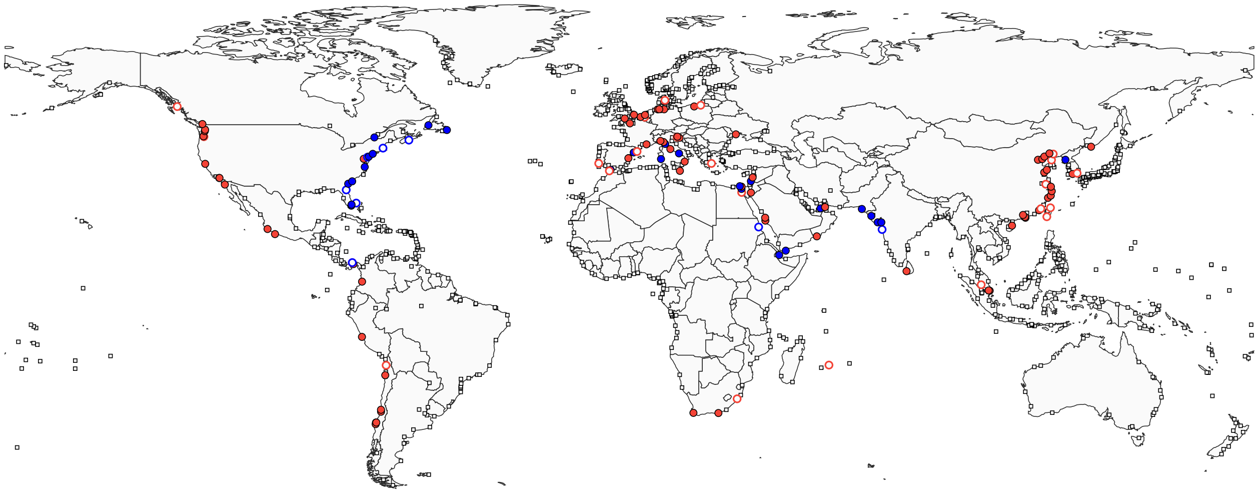

(c) Sample 6

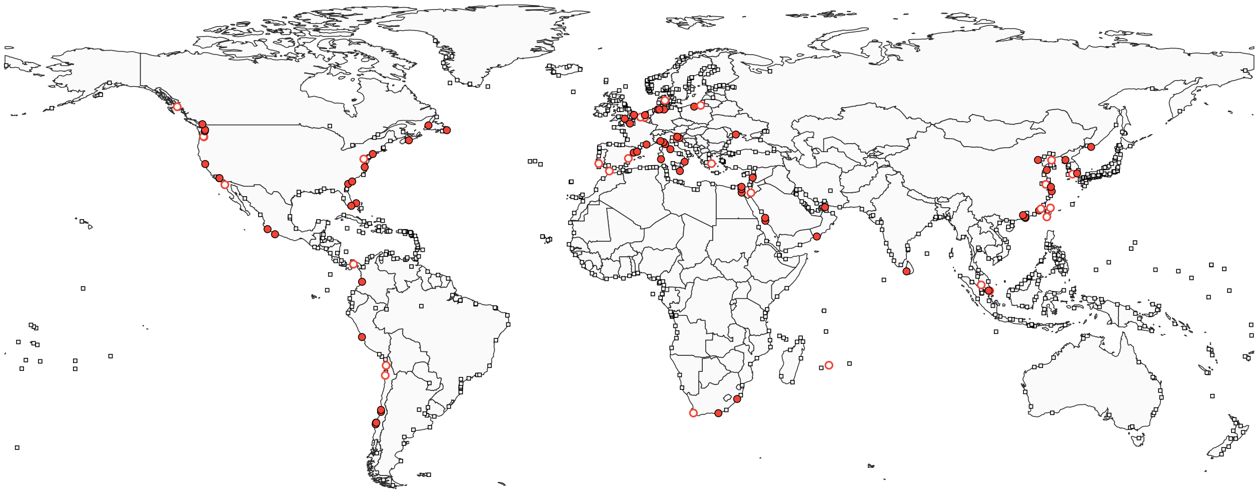

Supplementary Figures S4 (continued). 4th–6th samples.

(a) Sample 1

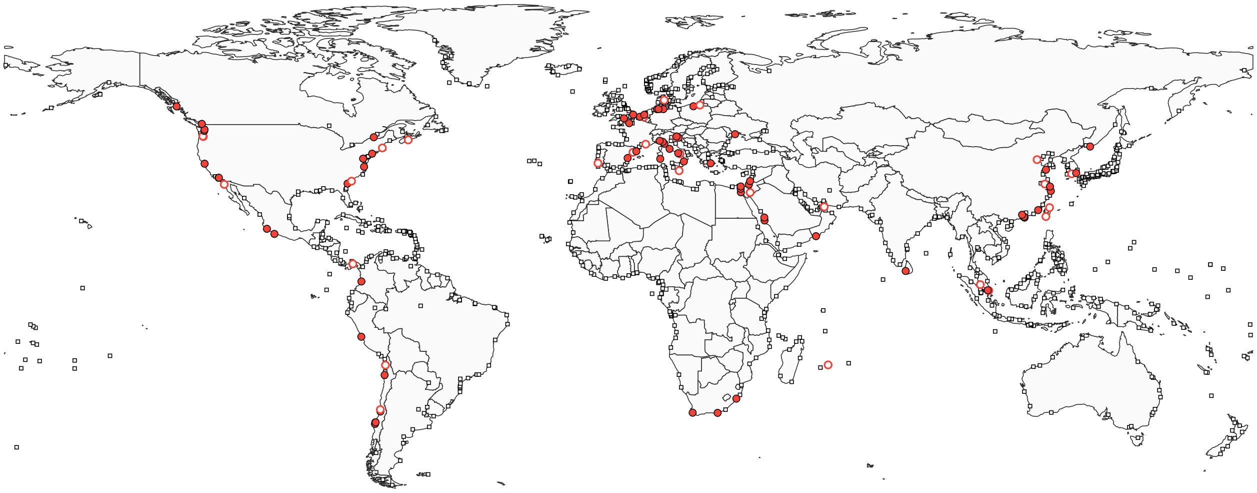

(b) Sample 2

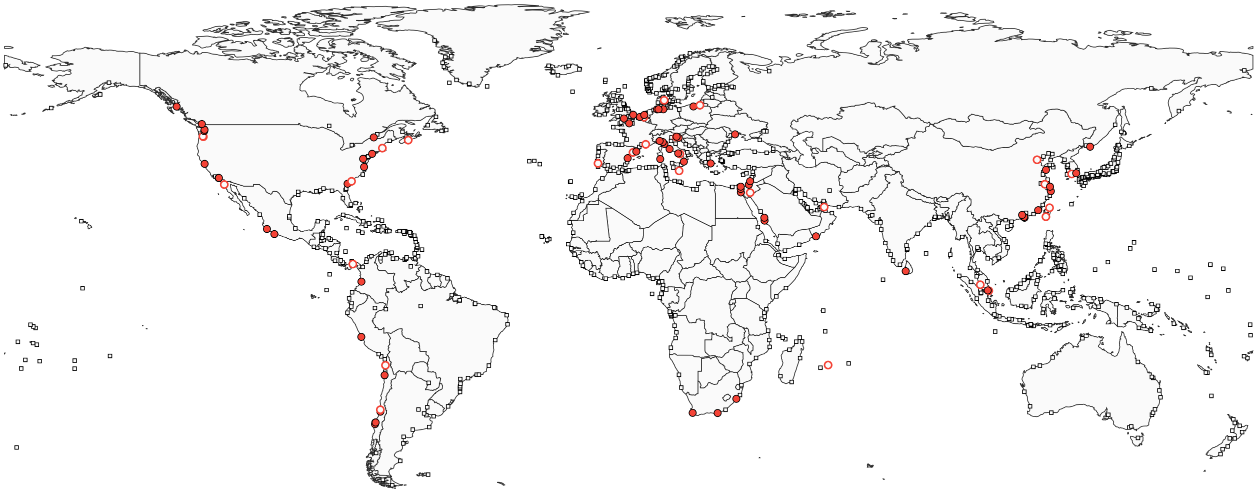

(c) Sample 3

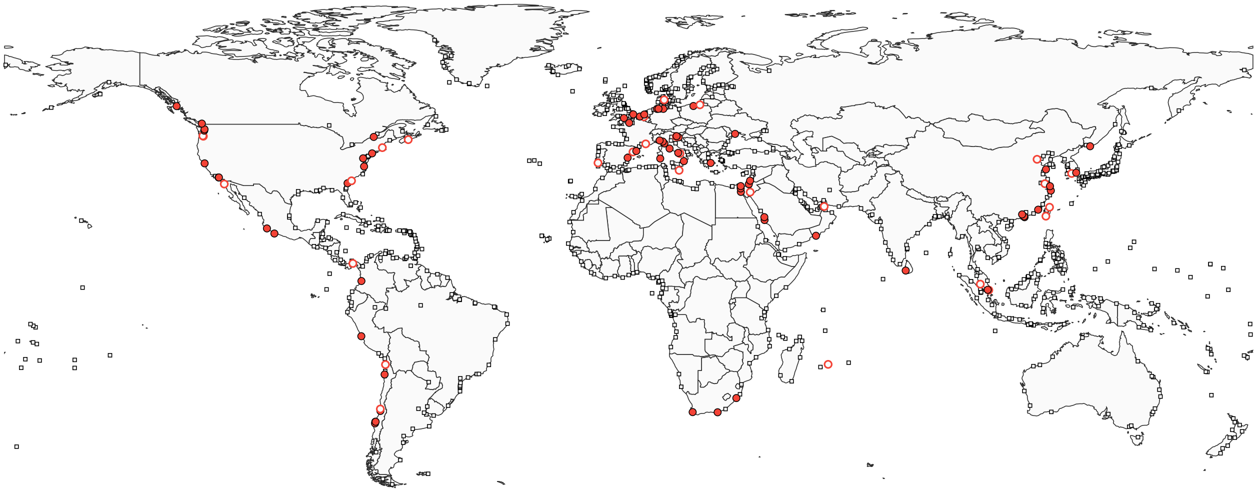

Supplementary Figure S5. CP structure detected at resolution $\gamma = 2.1$ (1st–3rd samples).

(a) Sample 4

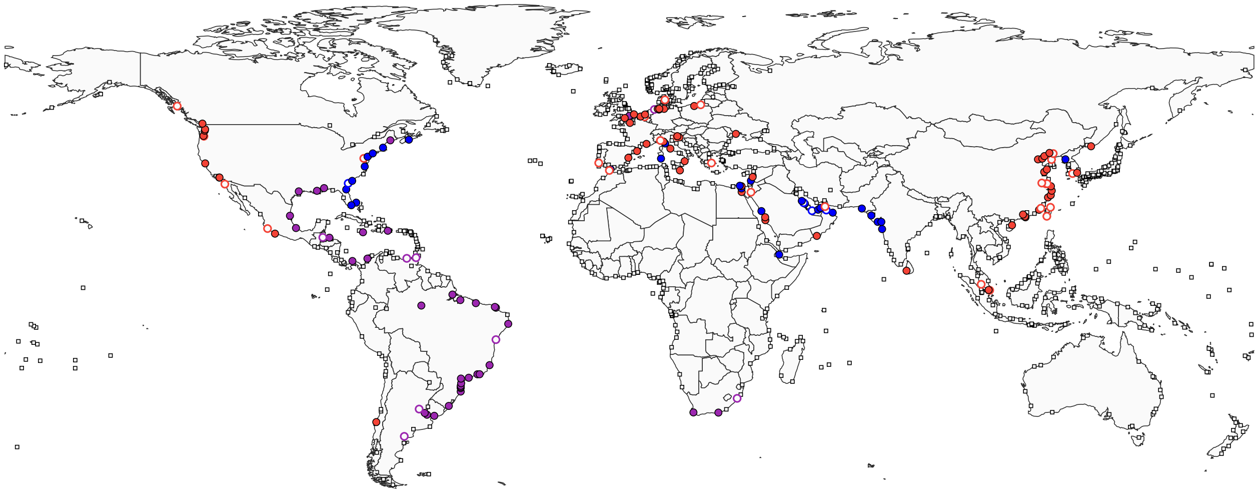

(b) Sample 5

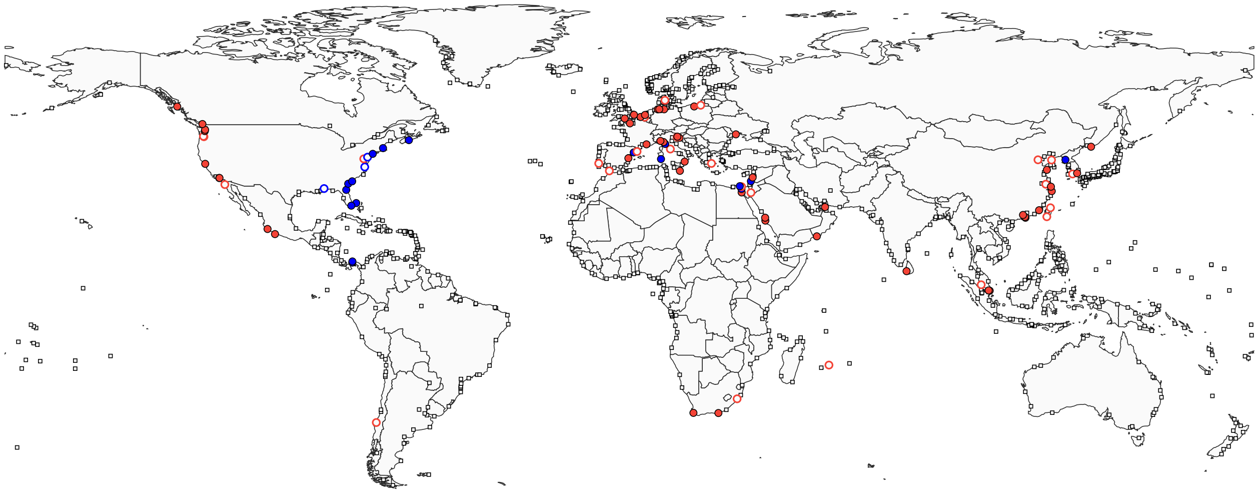

(c) Sample 6

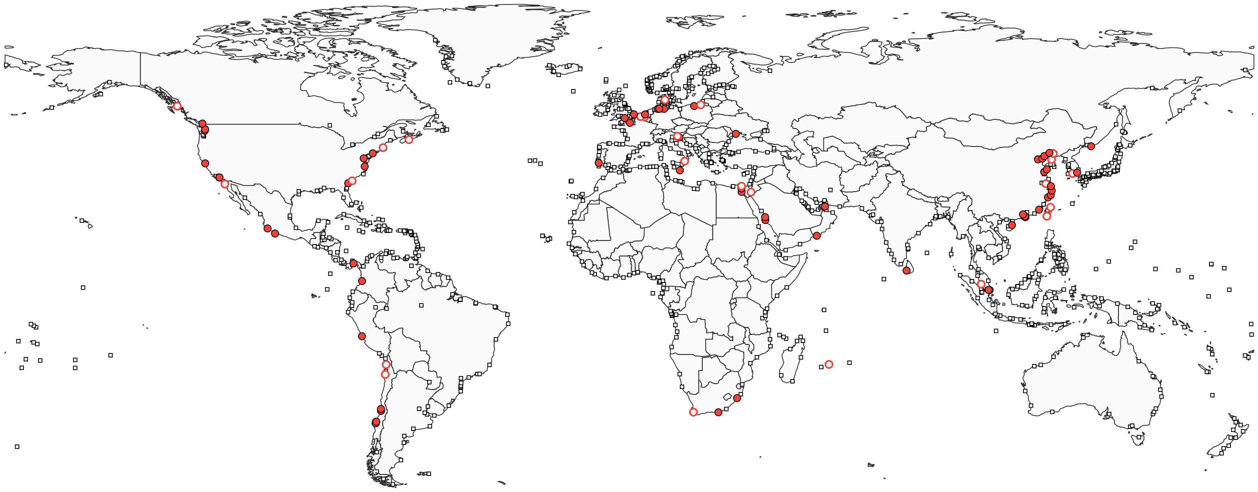

Supplementary Figures S5 (continued). 4th–6th samples.

(a) Sample 1

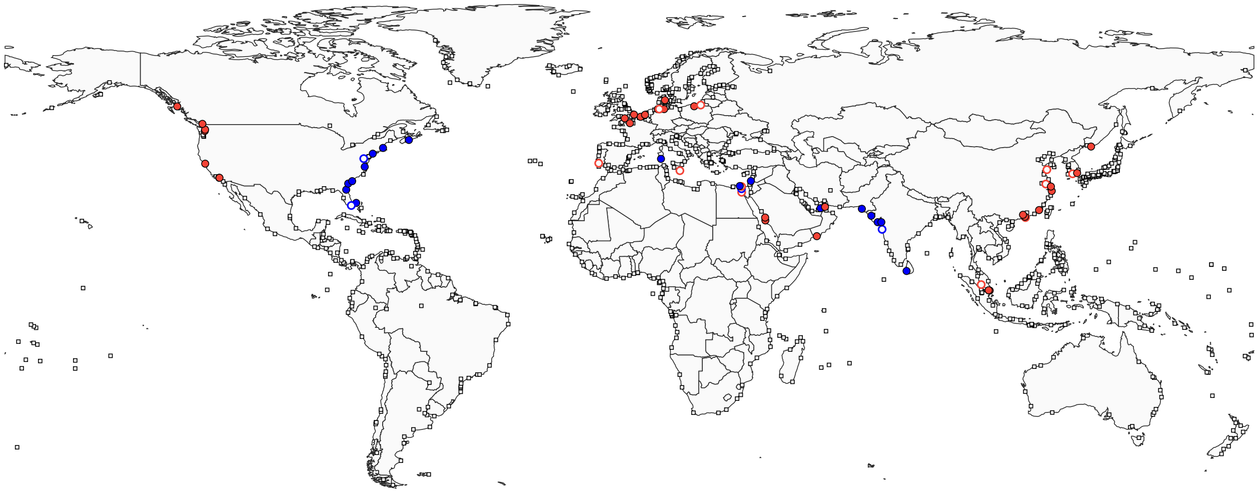

(b) Sample 2

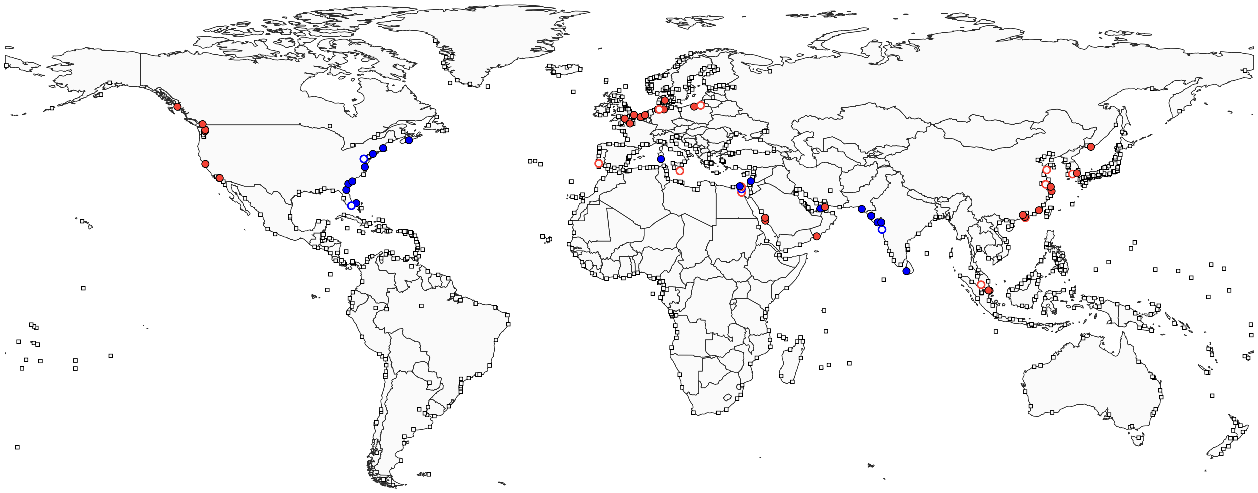

(c) Sample 3

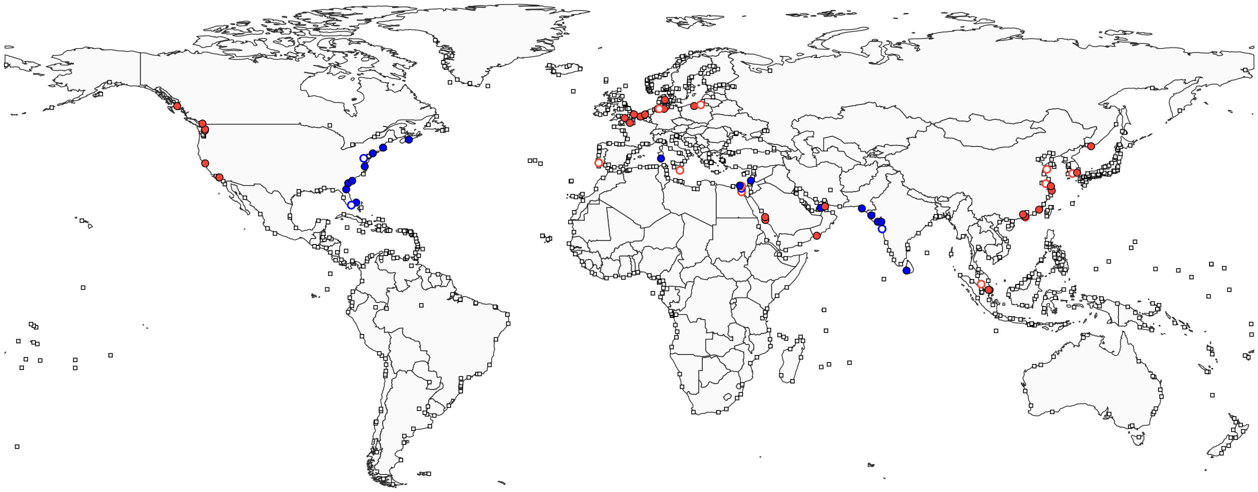

Supplementary Figure S6. CP structure detected at resolution $\gamma = 3.0$ (1st–3rd samples).

(a) Sample 4

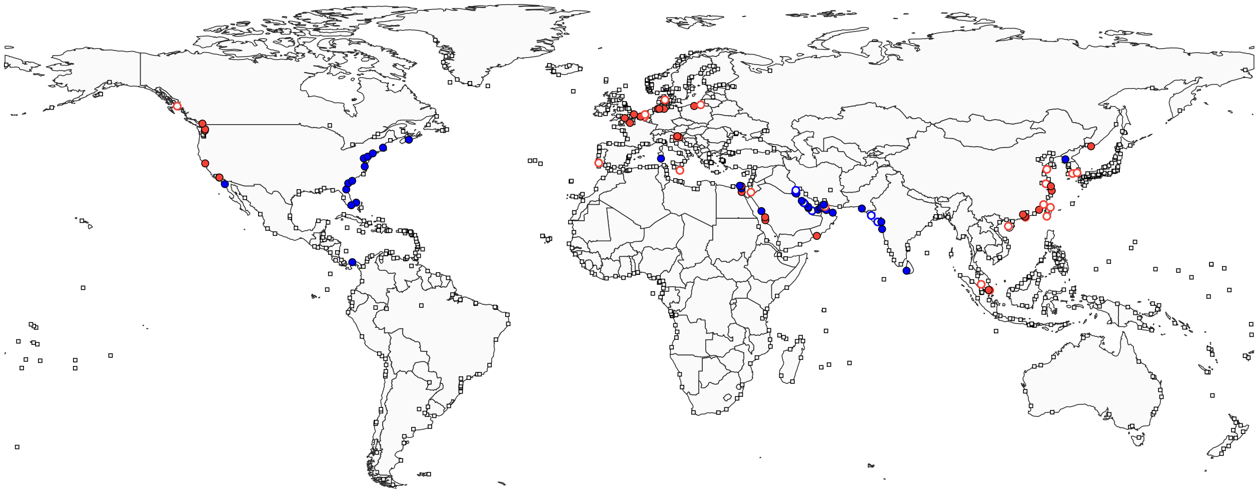

(b) Sample 5

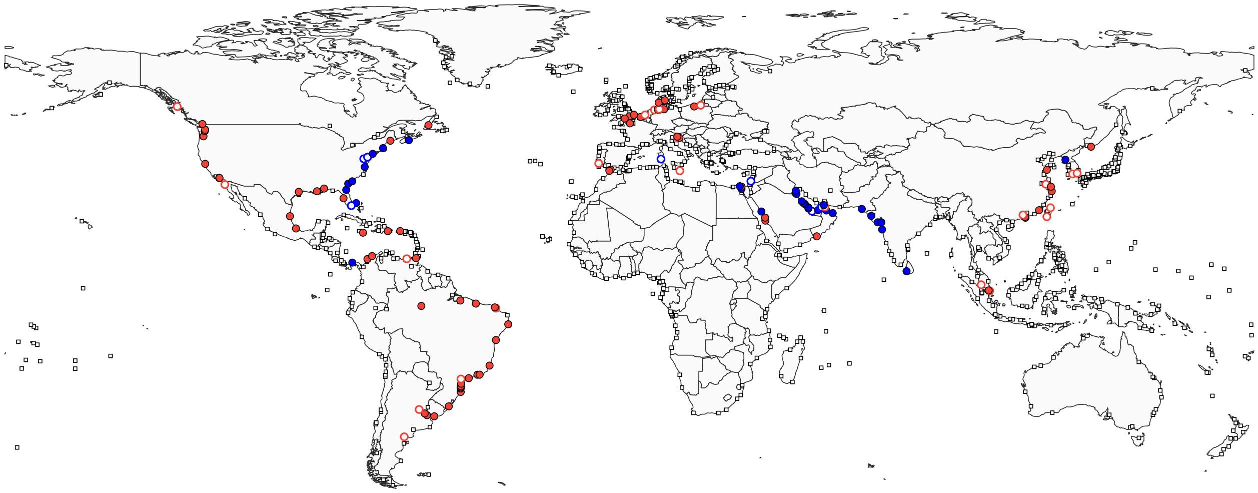

(c) Sample 6

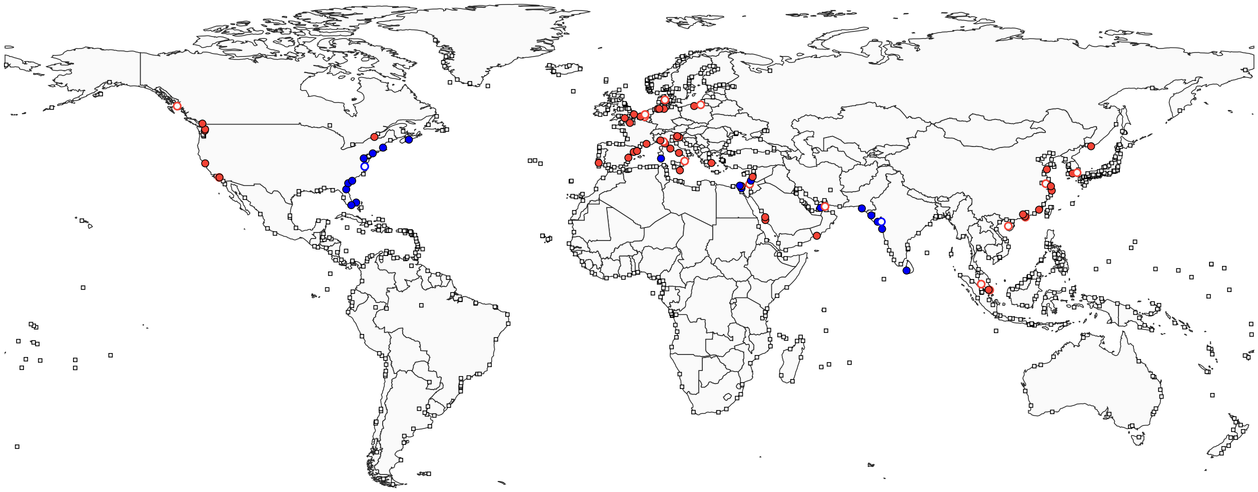

Supplementary Figures S6 (continued). 4th–6th samples.

(a) Sample 1

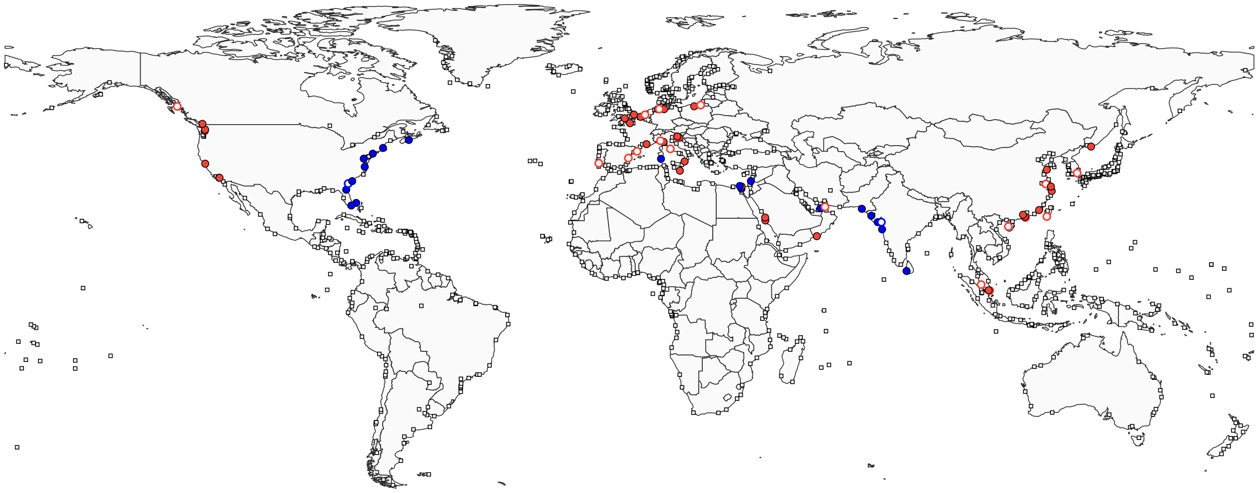

(b) Sample 2

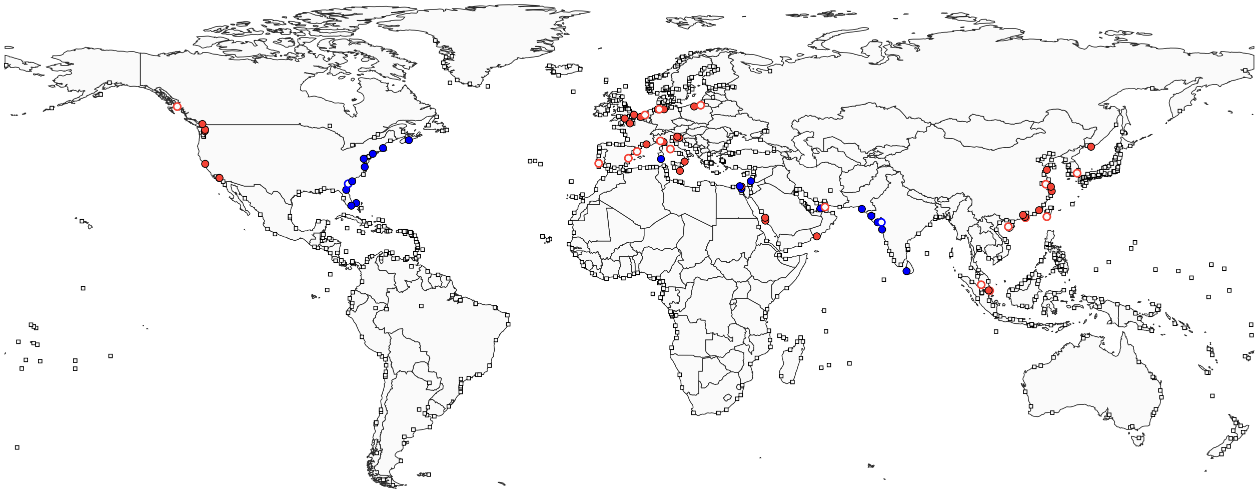

(c) Sample 3

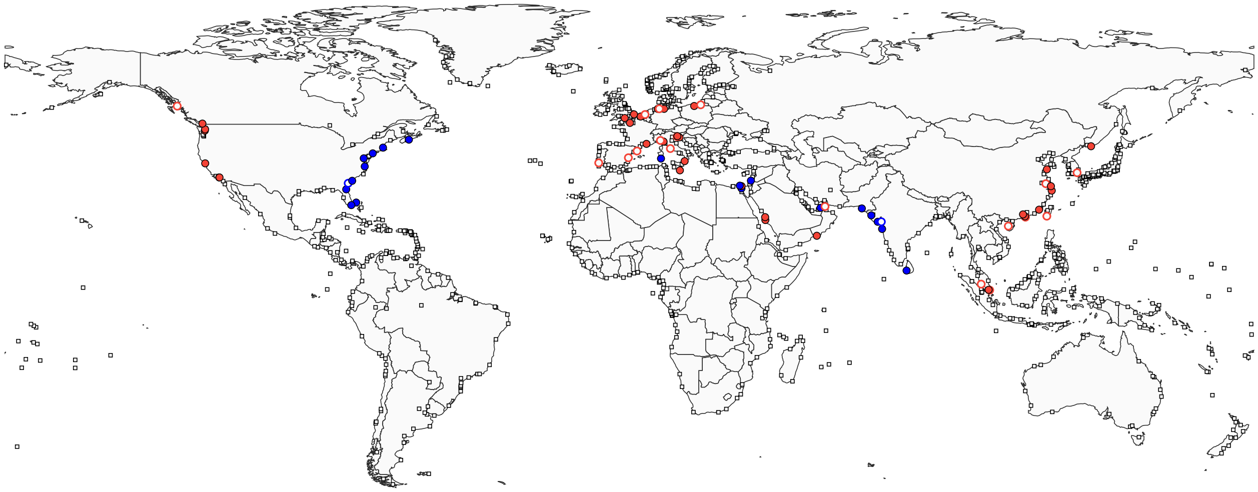

Supplementary Figure S7. CP structure detected at resolution $\gamma = 3.1$ (1st–3rd samples).

(a) Sample 4

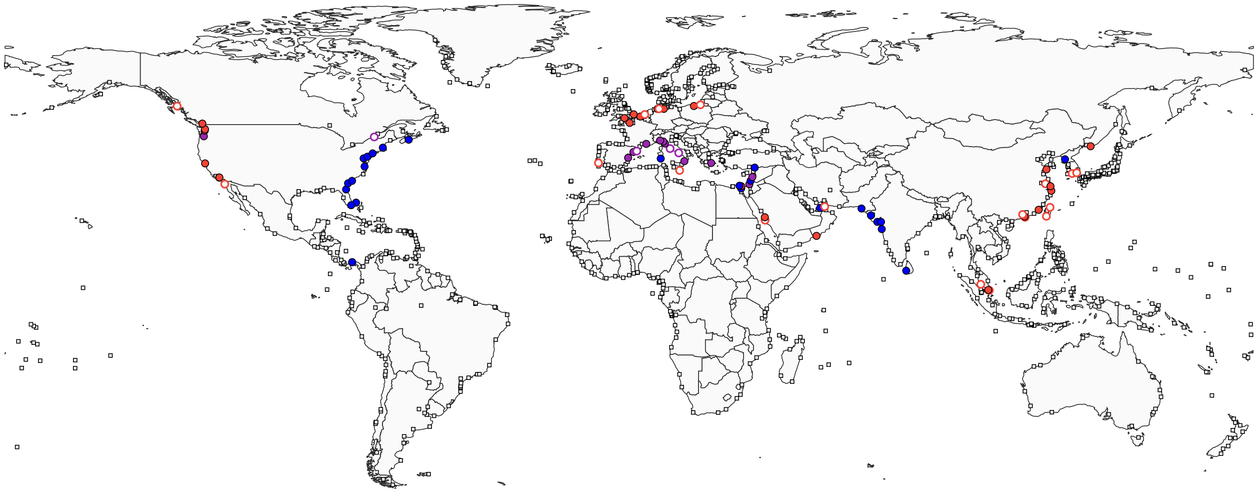

(b) Sample 5

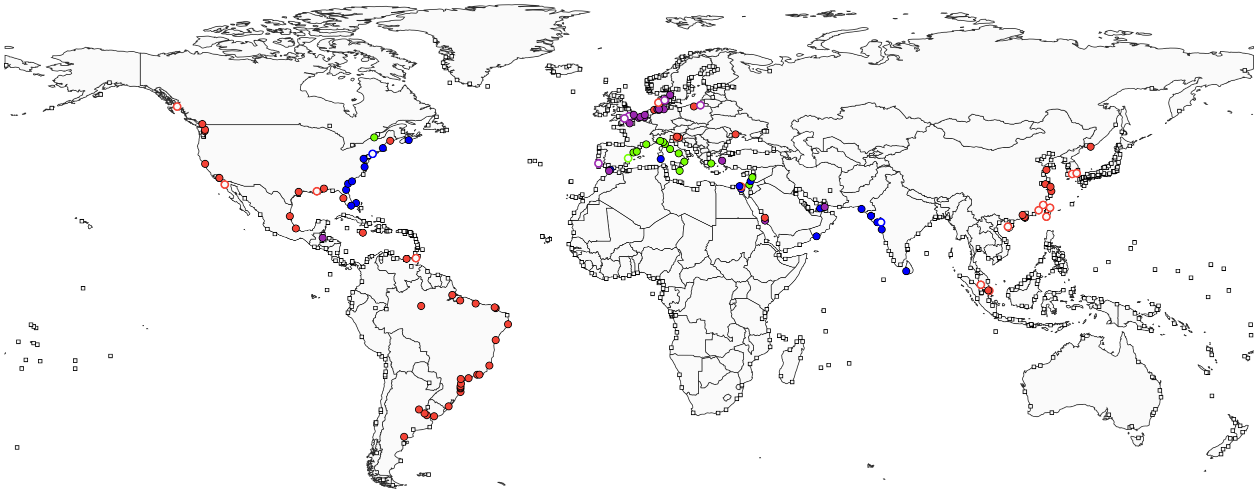

(c) Sample 6

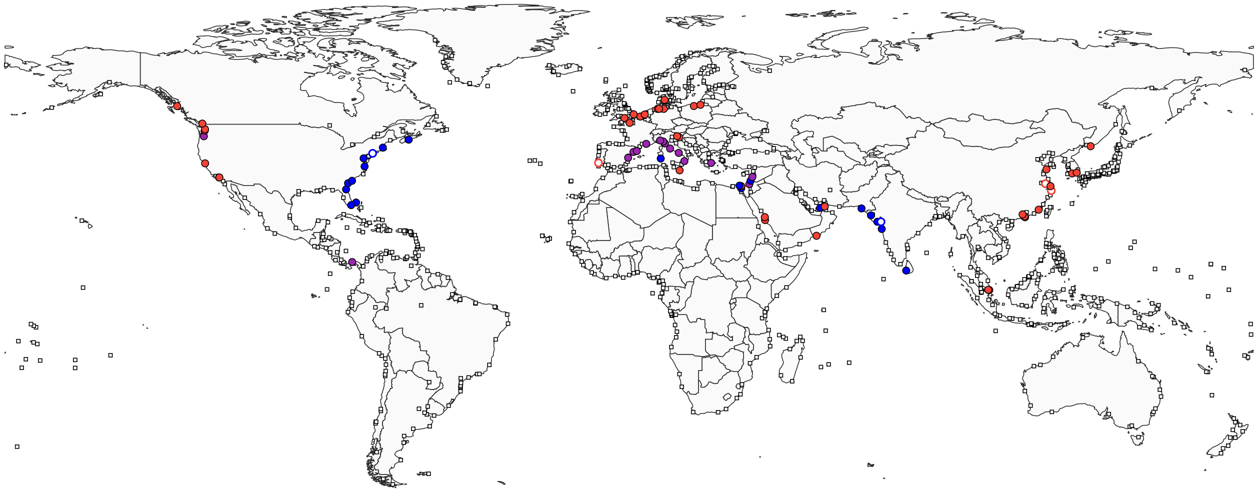

Supplementary Figures S7 (continued). 4th–6th samples.

(a) Sample 1

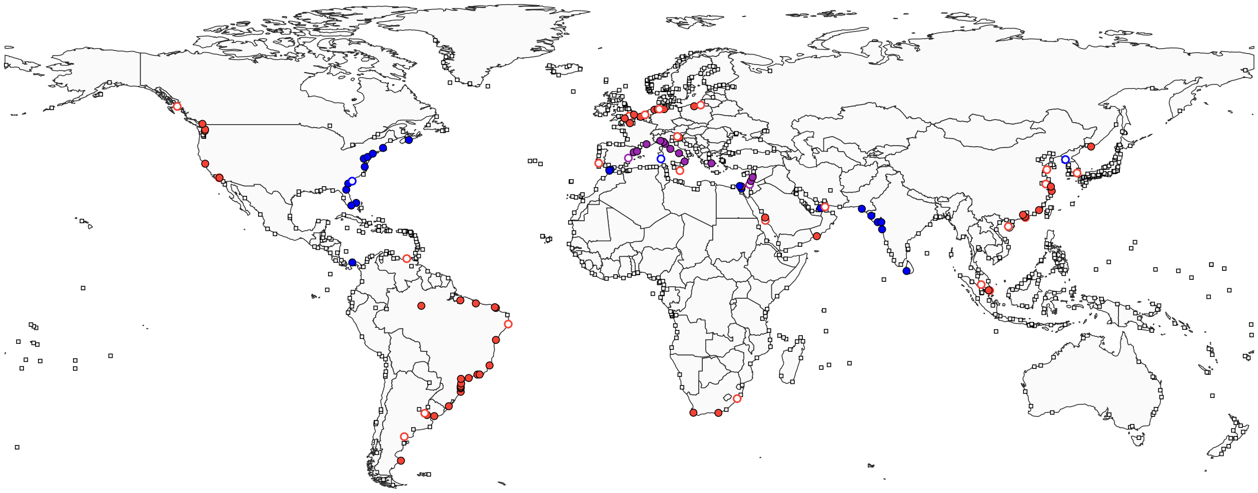

(b) Sample 2

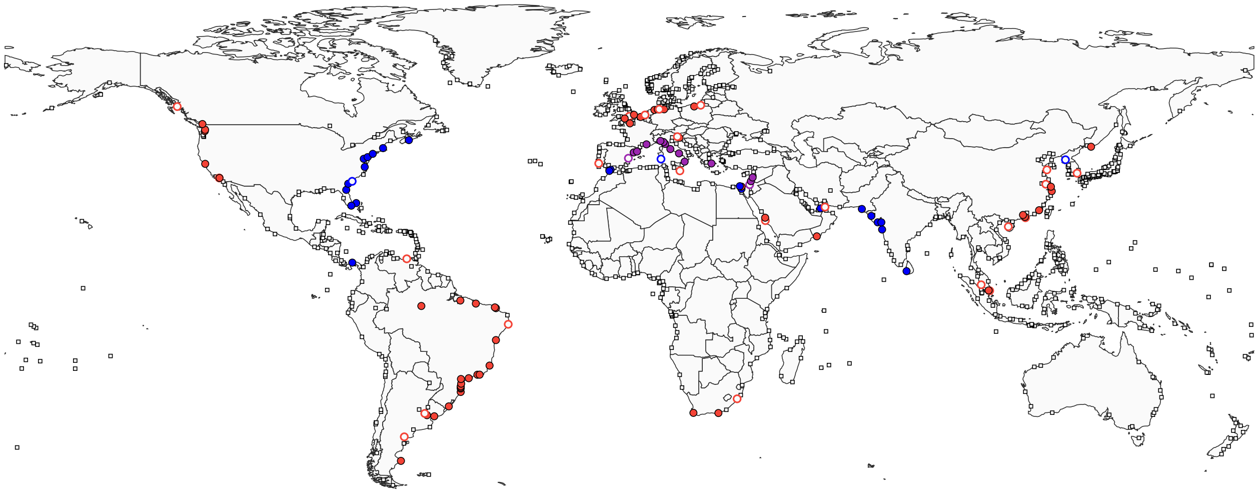

(c) Sample 3

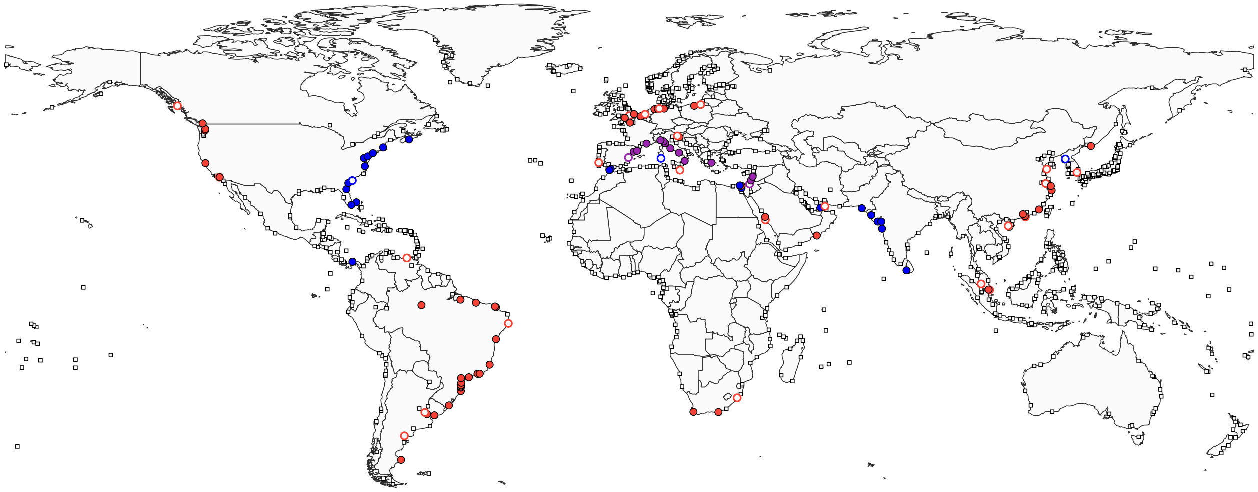

Supplementary Figure S8. CP structure detected at resolution $\gamma = 3.5$ (1st–3rd samples).

(a) Sample 4

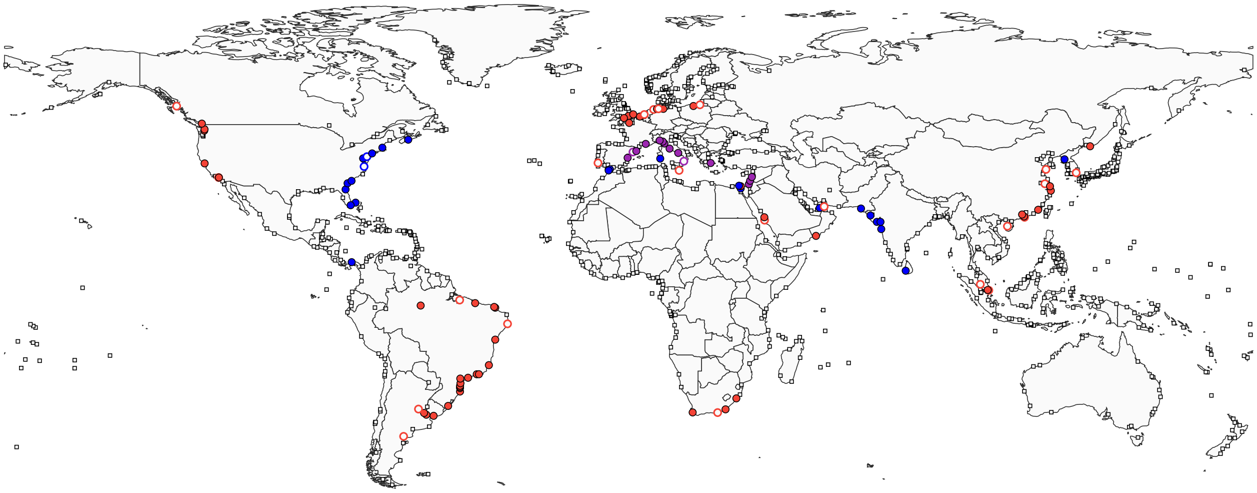

(b) Sample 5

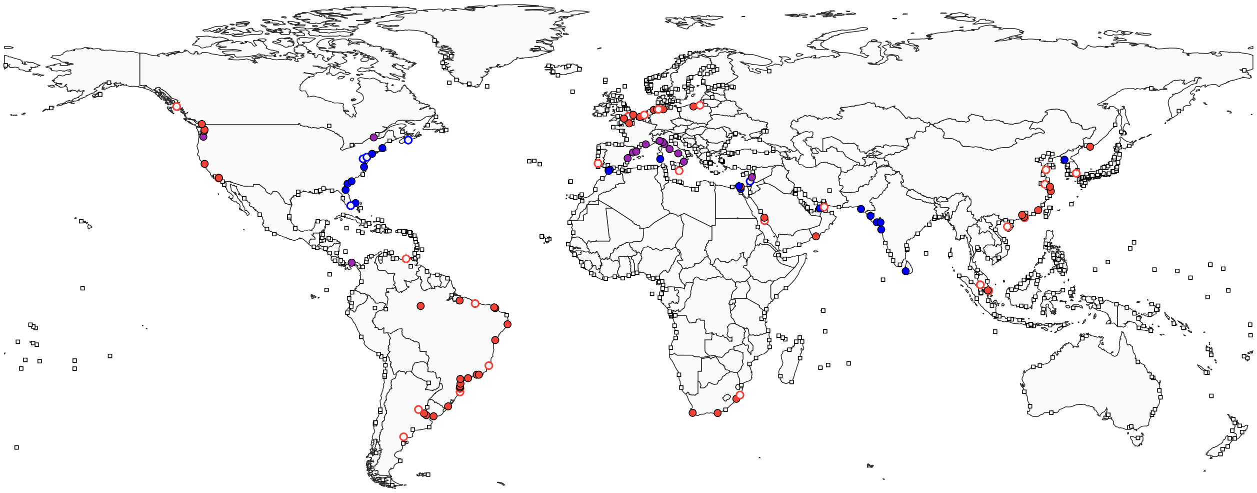

(c) Sample 6

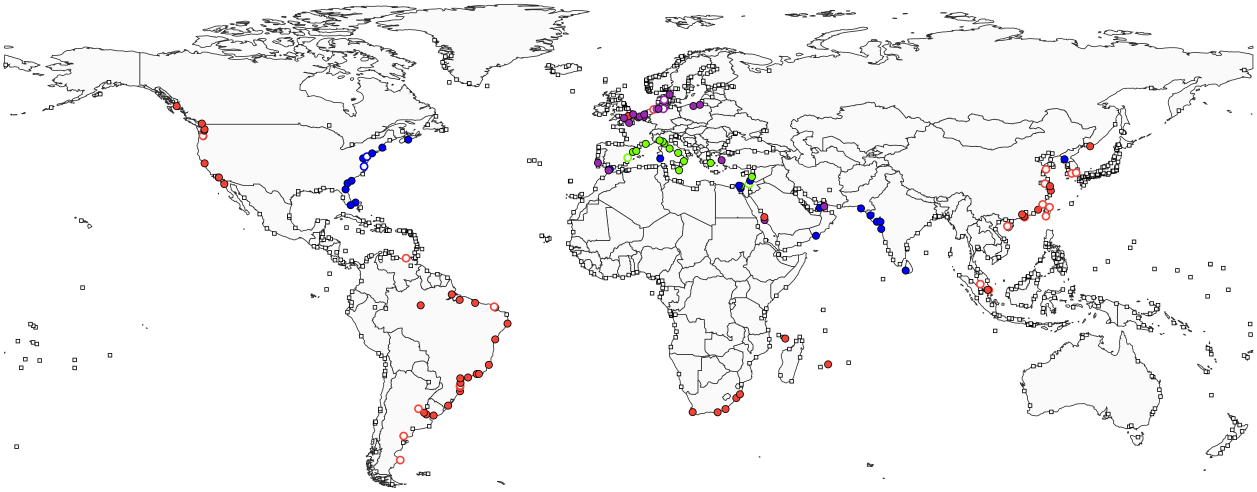

Supplementary Figures S8 (continued). 4th–6th samples.

(a) Sample 1

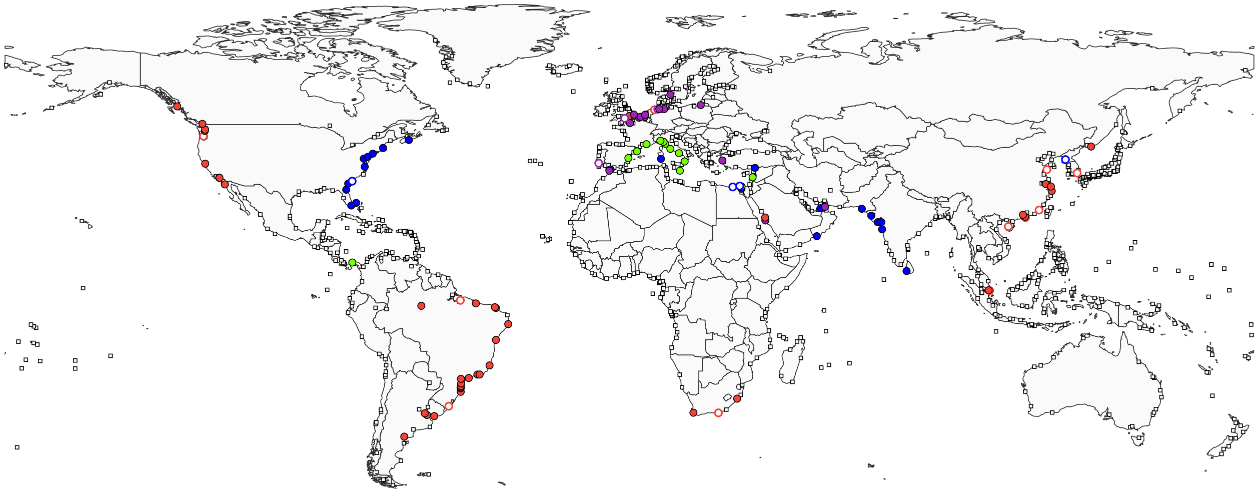

(b) Sample 2

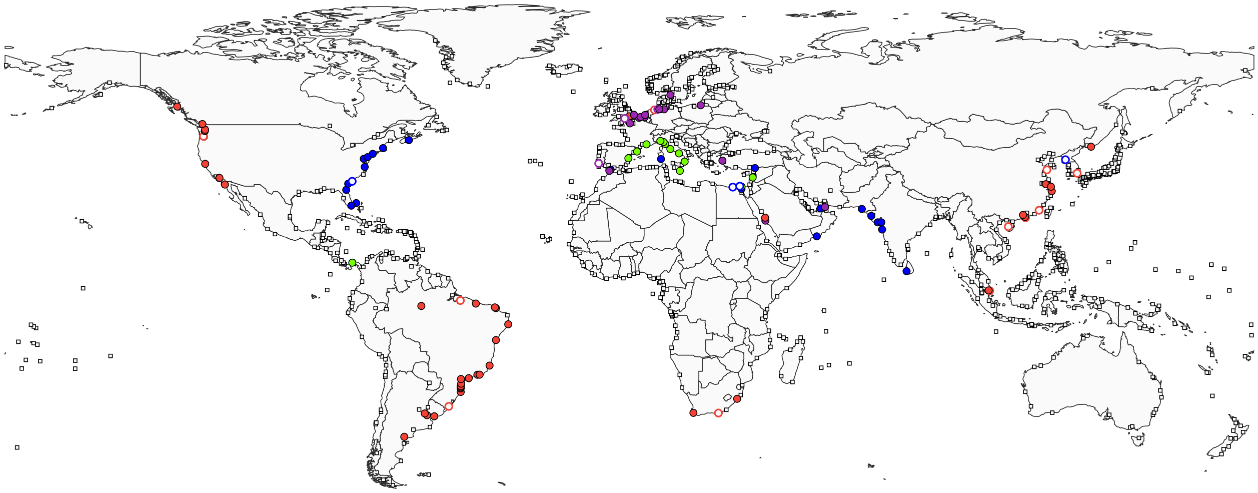

(c) Sample 3

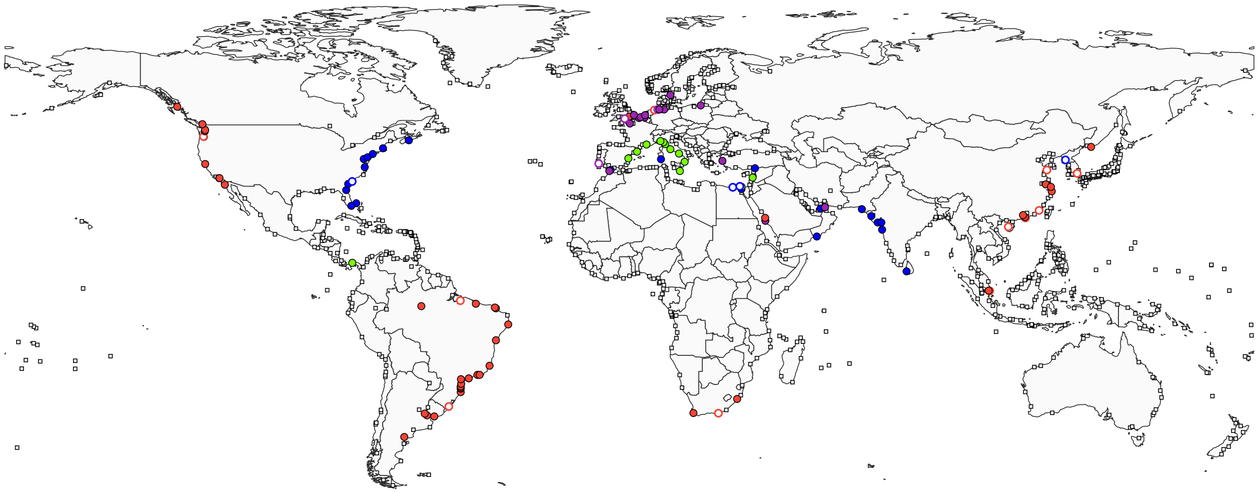

Supplementary Figure S9. CP structure detected at resolution $\gamma = 4.0$ (1st–3rd samples).

(a) Sample 4

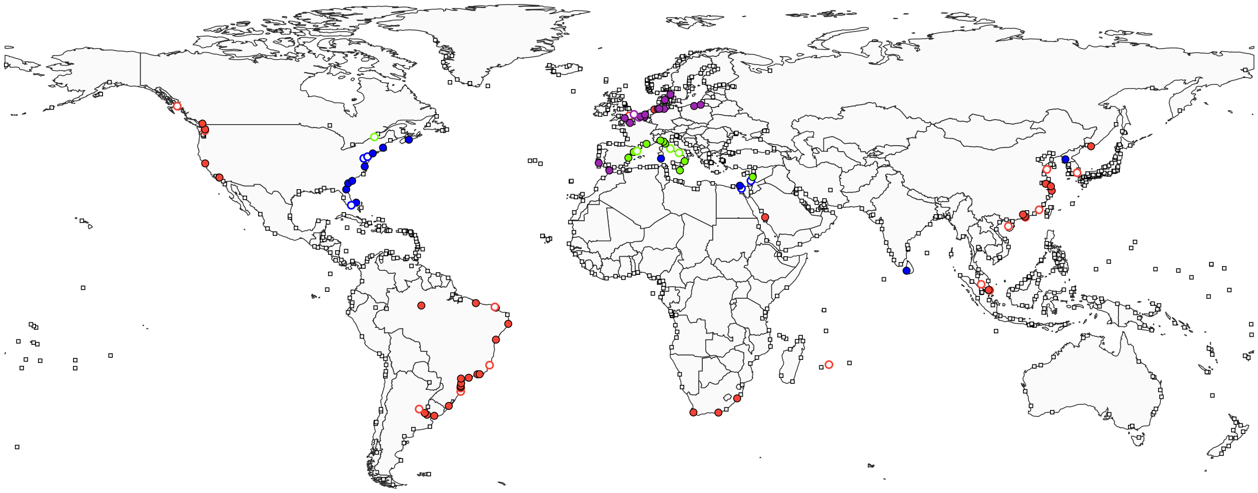

(b) Sample 5

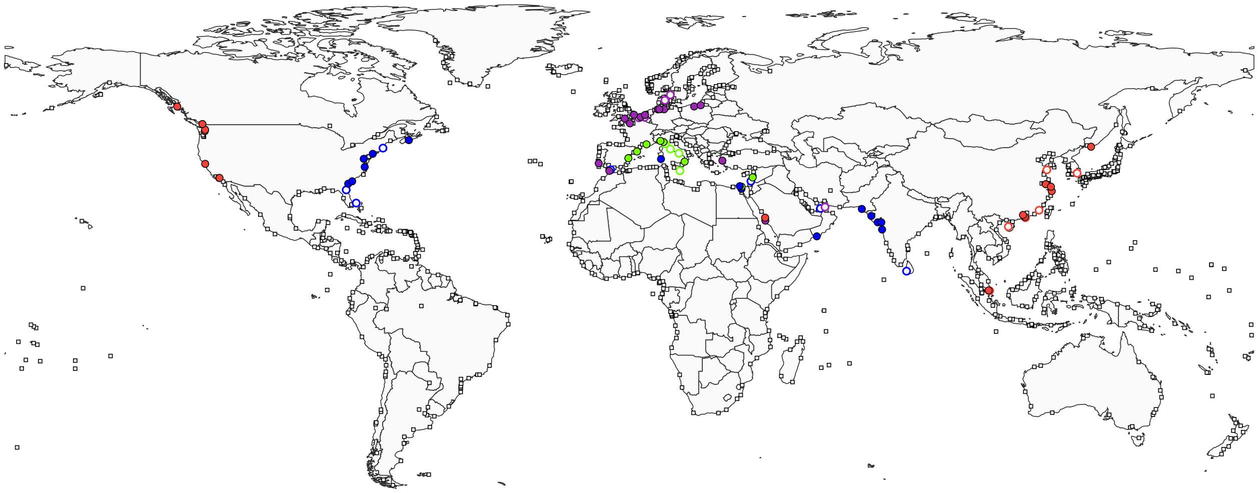

(c) Sample 6

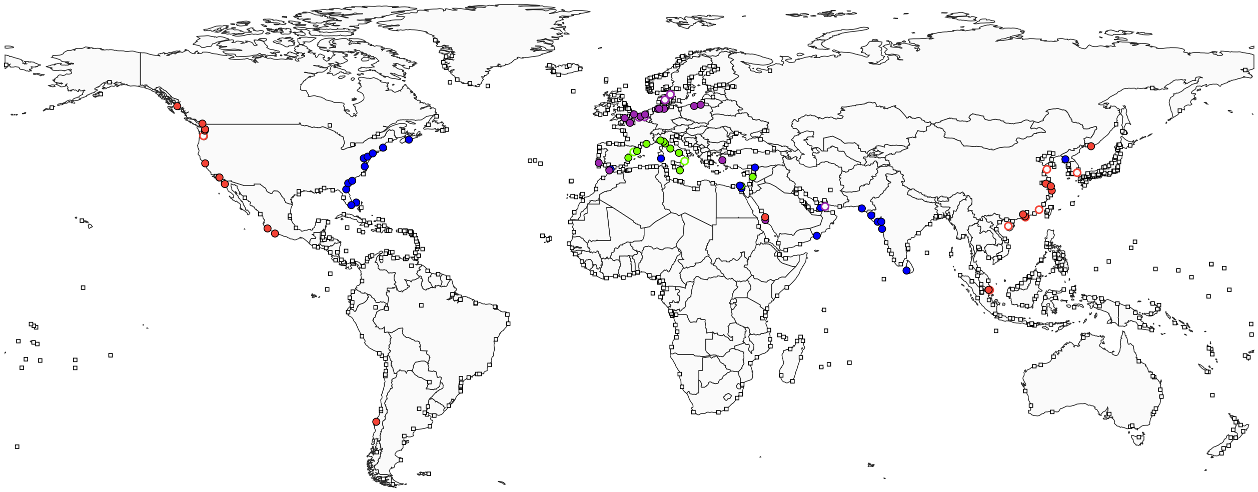

Supplementary Figures S9 (continued). 4th–6th samples.
